# A speed limit on serial strain replacement from original antigenic sin

**DOI:** 10.1101/2024.01.04.574172

**Published:** 2024-01-04

**Authors:** Lauren McGough, Sarah Cobey

**Affiliations:** 1 Department of Ecology and Evolution, The University of Chicago, Chicago, IL, 60637

## Abstract

Many pathogens evolve to escape immunity, yet it remains difficult to predict whether immune pressure will lead to diversification, serial replacement of one variant by another, or more complex patterns. Pathogen strain dynamics are mediated by cross-protective immunity, whereby exposure to one strain partially protects against infection by antigenically diverged strains. There is growing evidence that this protection is influenced by early exposures, a phenomenon referred to as original antigenic sin (OAS) or imprinting. In this paper, we derive new constraints on the emergence of the pattern of successive strain replacements demonstrated by influenza, SARS-CoV-2, seasonal coronaviruses, and other pathogens. We find that OAS implies that the limited diversity characteristic of successive strain replacement can only be maintained if $R_{0}$ is less than a threshold set by the characteristic antigenic distances for cross-protection and for the creation of new immune memory. This bound implies a “speed limit” on the evolution of new strains and a minimum variance of the distribution of infecting strains in antigenic space at any time. To carry out this analysis, we develop a theoretical model of pathogen evolution in antigenic space that implements OAS by decoupling the antigenic distances required for protection from infection and strain-specific memory creation. Our results demonstrate that OAS can play an integral role in the emergence of strain structure from host immune dynamics, preventing highly transmissible pathogens from maintaining serial strain replacement without diversification.

Antigenically variable pathogens consist of immunologically distinct strains whose spatiotemporal dynamics depend on the pathogen’s capacity to diversify and on the extent to which host immune responses elicited by one strain protect against infection with other strains. This interdependence gives rise to qualitatively distinct patterns of diversity or strain structures [1–6]. Examples include pathogens with high antigenic diversity at the host population scale (e.g., *Neisseria meningitidis* [7–10], enteroviruses [11]) and others with lower antigenic diversity at any given time but fast turnover (e.g., influenza A in humans [12,13], seasonal coronaviruses [14], SARS-CoV-2 [15–17], and others [18]).

Successive strain replacement typifies the dynamics of many common respiratory pathogens, yet it only arises in transmission models in some conditions [1, 2, 6, 12, 12, 19–30]. Large individual-based models have found that low antigenic diversity and fast turnover result from low mutation rates and strong cross-immunity between strains [6,31]. Short-term strain-transcending immunity [12,27] and punctuated antigenic changes [19, 32] have been hypothesized to be essential for serial antigenic replacement. Intrinsic transmissibility also plays a role, with higher $R_{0}$ promoting frequent emergence of antigenically novel strains [30, 31]. From a theoretical standpoint, successive strain replacement corresponds to epidemic dynamics that admit traveling wave solutions, in which infections create immunity that pushes existing strains in a single direction in phenotypic space [24, 33–36]. These dynamics agree with representations of H3N2 antigenic evolution, with strains following a low-dimensional trajectory in a higher-dimensional antigenic space [13, 37].

A common assumption in these investigations is that individuals acquire immunity specific to any strain that infects them, regardless of infection history [3, 20, 22, 23, 33]. Decades of experimental and observational evidence of “original antigenic sin” (OAS) have demonstrated that secondary responses expand memory B cell responses that target previously encountered epitopes, limiting the generation of responses to new sites [38–49]. This is especially apparent in responses to less immunogenic influenza vaccines [42, 50, 51]. Proposed mechanisms for this limited response to new sites include clearance of antigen by preexisting immunity [52,53] and the reduced requirements for memory compared to naive B cell activation (e.g., [54–56]). A straightforward corollary is that no protection can be derived from nonexistent responses to new sites, and thus individuals infected or vaccinated with the same strain may be differently protected to other strains, depending on whether they target conserved epitopes [57].

OAS might sometimes boost antibody responses to a site that are cross-reactive, blunting new responses to that site, but are not necessarily so protective. Decoy non-neutralizing epitopes are a feature of some pathogens [58, 59], but in influenza, this discrepancy between reactivity and protection appears to arise occasionally from OAS. For instance, middle-aged adults infected with H3N2 sometimes boost antibody titers that bind well by ELISA but show negligible neutralization activity against the infecting strain [60]. Ferrets infected with one influenza A subtype boost their stalk antibodies to that subtype on later infection with another subtype, blunting new responses [61]. The same pattern appears in children infected with H1N1 before H3N2 [61]. These recalled antibodies appear to be very poor binders to the second subtype, and although the in vivo consequences of hundreds-fold reduced affinity are uncertain [62], it is plausible they might be accompanied by reduced protection, as suggested by experimental infections in mice [63]. Thus, OAS might influence the protection derived from secondary exposures in two ways: first, by limiting any response to new sites, and second, by recalling memory responses to new but similar sites that poorly bind and weakly neutralize the new site. Either mechanism can in theory prevent the creation of protective memory to the infecting strain.

It is not known how OAS might affect pathogen evolution. Transmission models that include OAS predict “immune blind spots” in different age groups [64], which might relate to recent evidence of ∼24-year cycles in the induction of strong antibody responses to H3N2 [65]. Transmission models have assumed that only one strain circulates at any time and always mutates between seasons [64, 66], and thus have not addressed how OAS impacts antigenic evolution.

We investigate the consequences of OAS for pathogen evolution, and specifically for the conditions that permit successive strain replacement. Our mathematical models of host-pathogen coevolution incorporate OAS via the mechanisms described above. Both models allow for infection without creation of strain-specific memory through tunable parameters that allow cross-reactive responses to blunt the generation of new ones without conferring protection at that epitope. One model assumes a cross-reactive response at one epitope is sufficient to block the induction of new memory to the rest, and the other assumes that any epitope that is sufficiently diverged will elicit a new response. Nature is likely somewhere in between, depending on the type and number of secondary exposures [53, 63, 67, 68]. We show that these contrasting assumptions do not change our main result: OAS implies an upper bound on the reproduction number $R_{0}$ of pathogens that can exhibit successive strain replacement, and this bound implies limits on the speed of evolution, the standing antigenic diversity, and the time to the most recent common ancestor in this regime. OAS thus narrows the conditions in which serial strain replacement is likely to occur.

## Model

We separately consider the dynamics of protection and memory creation in individuals before describing pathogen dynamics in the host population.

### Host-scale protection

Pathogen strains and strain-specific memories are parametrized as real vectors in an abstract, continuous *d*-dimensional antigenic space in which the distance between two points represents the extent to which a strain-specific memory at one point protects against infection with a strain at the other point, as detailed mathematically below. The model assumes that each of the d dimensions of antigenic space corresponds to a physical region bound by antibodies, and the footprints of antibodies binding this region are entirely contained within it. We refer to these antibody-binding regions as epitopes. A similar model with each axis corresponding to one epitope was used in [69].

Individuals infected with a strain of the pathogen are also infectious. An infected individual can transmit either the infecting strain or a mutant strain. Infected individuals expose on average $R_{0}$ other individuals during their infection, where $R_{0}$ is the intrinsic reproduction number and does not vary by strain. The duration of infection also does not vary by strain.

Individuals are born without immunological memory. A naive individual always becomes infected when exposed to a strain and forms strain-specific memory on infection. A non-naive individual who becomes infected might develop memory specific to that strain. The set 𝒮 of strains $\vec{s}$ to which an individual has strain-specific memory at time t is their memory profile at t. An individual has at most one specific memory targeting (i.e., derived from infection with) each strain. The number of strains in 𝒮 equals m. In general, the number of strains in memory, m, would be expected to vary among individuals, though the population-scale model will assume that a fixed value of m is a reasonable approximation for computing protection.

**Figure 1:**
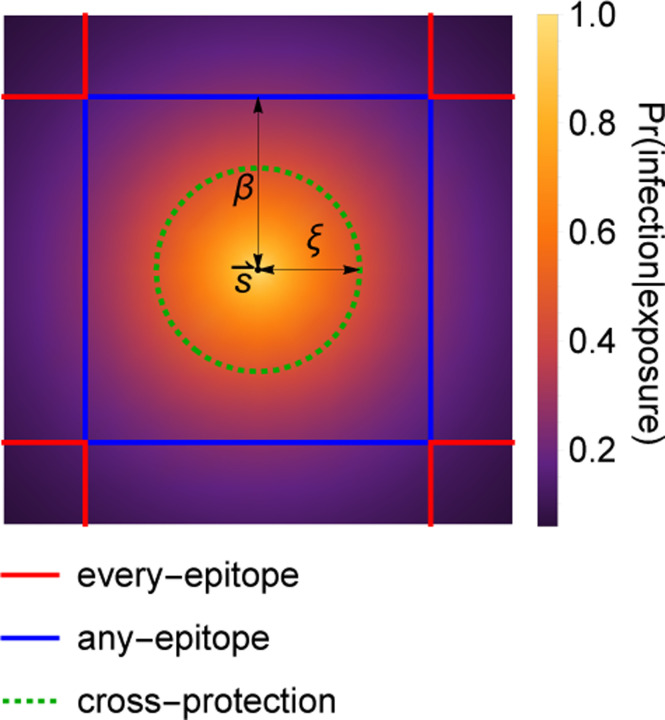
Cross-protection and blunting from a single memory in 2-dimensional antigenic space. A strain-specific memory $\vec{s}$ at the center protects against infection by nearby strains according to Eq. 1. The distance over which protection drops by a factor of 1/e is ξ, indicated in dotted green; the blunting distance is β; red lines are the outer boundary of the cross-shaped every-epitope model excluded region; blue lines are the outer boundary of the square any-epitope model excluded region. Only infections with strains outside these regions would generate new memory. Here, β=1.7 and ξ=1.25.

Memory decreases the probability of infection on exposure. Individuals cannot be reinfected by strains in their memory profile. Memory is cross-protective, meaning that strain-specific memory decreases the probability of infection with similar strains (Fig. 1). Cross-protection declines exponentially with the Euclidean antigenic distance between the strain in memory $\vec{s}$ and the challenge strain ${\vec{s}}^{\prime }$,

(1) $$\begin{array}{l} \mathrm{Pr}\left(\text{memory at }\vec{s}\text{ protects host }|\text{ host is exposed to }{\vec{s}}^{\prime }\right)\\ \;=e^{-\left\|\vec{s}-{\vec{s}}^{\prime }\right\|/\xi } \end{array}$$

with $\left\|\vec{s}-{\vec{s}}^{\prime }\right\|={\left(\sum_{i=1}^{d}{\left(s_{i}-s_{i}^{\prime }\right)}^{2}\right)}^{1/2}$. The quantity ξ is the cross-protection distance. It is an independent parameter that determines how antigenic change affects the protectiveness of existing memory to related strains. If ξ is high, cross-protection is high, and large mutations in antigenic space are required for protection to drop.

Eq. 1 defines the probability that a single strain-specific memory protects against infection by another strain. Each strain $\vec{s}$ in an individual’s memory profile 𝒮 contributes to protection against a challenge strain ${\vec{s}}^{\prime }$. The probability of infection for an individual with memory profile 𝒮 exposed to a challenge strain ${\vec{s}}^{\prime }$ is given by the product of the probabilities of each memory separately failing to protect,

(2) $$\mathrm{Pr}\left(\text{infection at }{\vec{s}}^{\prime }|\text{exposure to }{\vec{s}}^{\prime }\right)=\prod_{\vec{s}\in 𝒮}\left(1-e^{-\left\|\vec{s}-{\vec{s}}^{\prime }\right\|/\xi }\right)$$

Implicit in Eq. 2 is the assumption that protection against infection depends on the unordered set of strains in the memory profile, yet we have claimed that OAS induces a dependence on the order of exposure. Order dependence arises from the dynamics of memory creation.

### Creation of new memory

Infections and vaccinations do not always generate new specific memory to the challenge strain. Whether an individual generates a strain-specific memory depends on their existing memory profile. We model OAS via blunting, and consider two models, the “every-epitope” model and the “any-epitope” model, corresponding to the limits of biologically plausible scenarios. In each, a blunting distance, β, sets a threshold antigenic distance for developing new memory.

In the every-epitope model, the challenge strain ${\vec{s}}^{\prime }$ must escape immunity at every epitope to create strain-specific memory (Fig. 1); escape at only some epitopes can be overcome by adaptive immune responses, including antibodies binding conserved regions [69]. More precisely, an individual challenged with a strain ${\vec{s}}^{\prime }$ creates strain-specific memory if ${\vec{s}}^{\prime }$ differs from every strain $\vec{s}$ in their memory profile 𝒮 by an antigenic distance of at least β in each of its d antigenic space coordinates,

(3) $$\begin{array}{l} \text{every-epitope condition for memory creation}:\\ \;\underset{\vec{s}\in 𝒮}{\min}\underset{i=1,\ldots ,d}{\min}\left|s_{i}^{\prime }-s_{i}\right|\geq \beta . \end{array}$$

In the any-epitope model, it is enough for the challenge strain ${\vec{s}}^{\prime }$ to escape immunity in a single epitope for each $\vec{s}$ to create strain-specific memory (Fig. 1) [3]. An individual challenged with a strain ${\vec{s}}^{\prime }$ creates strain-specific memory if ${\vec{s}}^{\prime }$ differs from every strain $\vec{s}$ in their memory profile 𝒮 by at least β in at least one epitope (which may not be the same epitope across 𝒮),

(4) $$\begin{array}{l} \text{any-epitope condition for memory creation}:\\ \;\underset{\vec{s}\in 𝒮}{\min}\underset{i=1,\ldots ,d}{\max}\left|s_{i}^{\prime }-s_{i}\right|\geq \beta . \end{array}$$

These models can be conceptualized as different types of exposures. The every-epitope model could represent an exposure to a small dose of antigen, for example via some natural infections, where the presence of any neutralizing memory can limit pathogen replication, reducing antigen availability enough to prevent the generation of new memory. The any-epitope model could represent an exposure to a high dose of antigen or an immunogenic vaccine that forces a response to diverged epitopes even in the presence of memory that would ordinarily suppress a new response. This effect has been shown in animal models since the earliest observations of OAS [46], and more recently in molecular detail in mice [48, 68].

### Population-scale infection-immunity coupling

At the population scale, our model is equivalent to the model of pathogen-immune coevolution developed in [36] with the added assumptions that each dimension corresponds to an epitope in the proper coordinates and that over the timescales of interest, each epitope evolves at a fixed rate. The population-scale description is formulated in terms of two densities in antigenic space: the total number of individuals infected by each strain $\vec{s}$ at time t and the total number of individuals with strain-specific memory to $\vec{s}$ at time t, denoted $n\left(\vec{s},t\right)$ and $h\left(\vec{s},t\right)$, respectively. In transitioning to a density-based approach, we trade complete information about each individual’s memory profile for tractability. This is similar to the choice of a previous cohort-level description for studying OAS [64], although the motivation is different as we do not incorporate age structure.

Strain fitness is determined by the rate of transmission per infectiousness period, which depends on individuals’ immunity. Given complete information about the memory profile of every individual, this probability would be an exactly computable average, but with only the density $h\left(\vec{s},t\right)$ available, we must make assumptions on how protection is distributed. We make a homogeneity assumption, that the average protection against infection across individuals is well approximated by the average protection in a system where the $h\left(\vec{s},t\right)$ memories are distributed randomly among individuals, with each individual having memory to a fixed number of strains, m. The homogeneity assumption precludes direct consideration of dynamics where the population consists of several groups of individuals with highly correlated infection histories that differ substantially between groups, such as birth cohorts; however, this kind of structure could be accommodated by introducing a separate memory density for each cohort and specifying transmission dynamics between cohorts.

The expected protection against infection upon exposure to strain $\vec{s}$ conferred by a single memory chosen uniformly at random from $h\left({\vec{s}}^{\prime },t\right)$ is denoted $c\left(\vec{s},t\right)$. Using the definition in Eq. 1, this is equal to

(5) $$c\left(\vec{s},t\right)=\frac{1}{mN_{h}}\int_{{\vec{s}}^{\prime }\text{ in antigenic space}}d{\vec{s}}^{\prime }\;h\left({\vec{s}}^{\prime },t\right)e^{-\left\|\vec{s}-{\vec{s}}^{\prime }\right\|/\xi },$$

where $N_{h}$ is the size of the population and the factor $mN_{h}$ adjusts for the normalization of $h\left({\vec{s}}^{\prime },t\right)$. If the average susceptibility to $\vec{s}$ across individuals in the population is well approximated by the susceptibility of an individual with m strains in its memory profile chosen at random from $h\left(\vec{s},t\right)$, then in analogy with Eq. 2, we have

(6) $$\mathrm{Pr}\left(\text{infection at }\vec{s}|\text{ exposure at }\vec{s}\right)\approx {\left(1-c\left(\vec{s},t\right)\right)}^{m}.$$

Defining $R_{0}$, the intrinsic reproduction number of the pathogen, to be the expected number of people an infected individual encounters during their infection (and would infect if they were susceptible), the fitness of strain $\vec{s}$ at time t is

(7) $$f\left(\vec{s},t\right)=\log\left(R_{0}{\left(1-c\left(\vec{s},t\right)\right)}^{m}\right),$$

as in [36].

We model the successive strain replacement regime using the traveling wave model described in [36]. Traveling wave solutions arise from stochastic coupled dynamical equations between the infection density and the memory density that take into account fitness differences among strains, mutations, noise in the transmission process, and memory addition and deletion:

(8) $$\partial_{t}n\left(\vec{s},t\right)=f\left(\vec{s},t\right)n\left(\vec{s},t\right)+D{\vec{\nabla }}^{2}n\left(\vec{s},t\right)+\sqrt{n\left(\vec{s},t\right)}\eta \left(\vec{s},t\right),$$

(9) $$\partial_{t}h\left(\vec{s},t\right)=n\left(\vec{s},t\right)-\frac{N_{I}\left(t\right)}{mN_{h}}h\left(\vec{s},t\right).$$

The first equation (Eq. 8) states that as the pathogen spreads, each strain grows or shrinks according to its fitness (Eq. 7); mutations are frequent and have small effect, such that the diffusion approximation is appropriate with diffusion constant D; and pathogen spread is stochastic, with Gaussian process noise, where $\eta \left(\vec{s},t\right)$ is a unit Gaussian $\left(\langle \eta \left(\vec{s},t\right)\rangle =0,\;\langle \eta \left(\vec{s},t\right)\eta \left({\vec{s}}^{\prime },t^{\prime }\right)\rangle =\delta \left(\vec{s}-{\vec{s}}^{\prime }\right)\partial \left(t-t^{\prime }\right)\right)$, and the prefactor $\sqrt{n\left(\vec{s},t\right)}$ sets the mean and standard deviation. The second equation (Eq. 9) states that the change in memory at a given time is determined by memory addition at the locations of infections and by memory deletion uniformly at random: each person acquires a new strain-specific memory at $\vec{s}$ when they are infected by strain $\vec{s}$, and at that time, they lose one memory from their memory profile 𝒮, such that each person maintains a fixed number of m memories over time. Time is modeled in units of the infectiousness period.

### Successive strain replacement dynamics

Successive strain replacement dynamics are conceptualized as an infection distribution that takes the form of a localized clump that maintains its shape as it moves through antigenic space at a constant speed and direction and is Gaussian in the direction of motion. The localization implies that strains are immunologically well-defined, with low antigenic diversity around any point at any time. The constant speed and direction amount to assuming that each epitope evolves at a constant rate, with negligible impact from stochasticity in the directions orthogonal to the direction of motion in which the wave travels.

Successive strain dynamics are most simply expressed in coordinates where one axis is aligned with the direction of motion, with the remaining axes aligned orthogonally. We use $\vec{x}$ (rather than $\vec{s}$) to indicate “wave-adapted” antigenic space coordinates where the $x_{1}$ axis lies along the axis of antigenic evolution.

**Figure 2:**
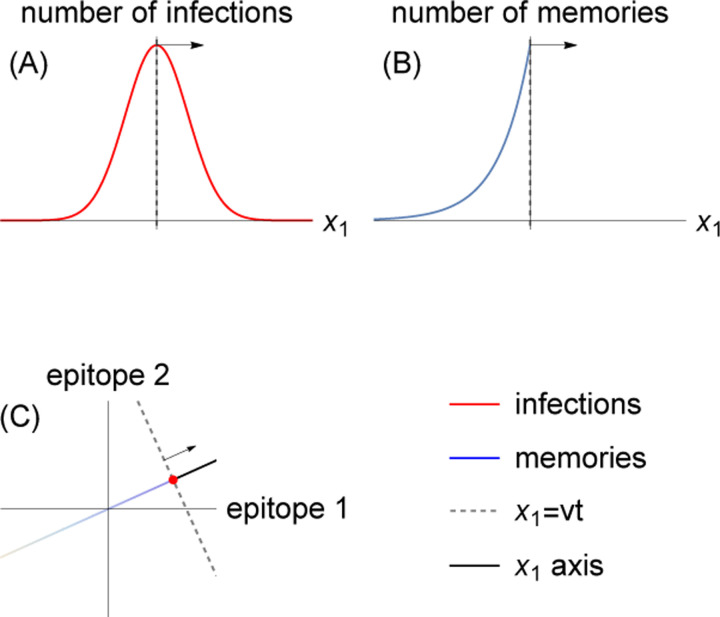
Successive strain replacement as traveling waves in antigenic space. (A) Strain replacement is modeled as a localized infection distribution moving along a one-dimensional trajectory in *d*-dimensional antigenic space with constant velocity $\vec{v}$. (B) The memory distribution is an exponentially decaying distribution with wavefront at $x_{1}=vt$, localized in orthogonal directions. (C) The axes aligned with the direction of motion of the infection and memory distributions are not the same as the epitope-aligned axes. The localized infection peak is visualized as a point at the location of the wavefront.

As in [36], the infection distribution takes the form

(10) $$n\left(\vec{x},t\right)=\frac{N_{I}}{{\left(2\pi \sigma^{2}\right)}^{1/2}}e^{-\frac{{\left(x_{1}-vt\right)}^{2}}{\sigma^{2}}}\rho \left(x_{2},\ldots ,x_{d}\right)$$

where $N_{I}$ is the number of infected individuals, v is the wave velocity, σ is the standard deviation of the Gaussian in the direction of motion, and ρ is a normalized distribution. Both the Gaussian and ρ are taken to be localized to a region of linear dimension much less than the cross-protection distance $\xi \left(\sigma \ll \xi \right)$ (Fig. 2A). We are interested in the case where the cross-protection distance is less than the blunting distance, and thus these are also localized to a region of linear dimension much less than the blunting distance $\beta \left(\sigma \ll \beta \right)$.

The memory distribution that couples to Eq. 10 is a wavefront with maximum at the peak of the infection distribution that falls off exponentially fast in the direction of earlier infections (Fig. 2B). In a zoomed-out limit where $x_{1}\gg \sigma$ and $x_{i}\gg \beta$ for i=2,…,d,

(11) $$h\left(\vec{x},t\right)\approx \frac{mN_{h}}{v\tau }e^{-\left(vt-x_{1}\right)/v\tau }\text{Θ}\left(vt-x_{1}\right)\delta \left(x_{2}\right)\cdots \delta \left(x_{d}\right),$$

where $N_{h}$ is the population size, $\tau =mN_{h}/N_{I}$, and Θ is a step function, $\text{Θ}\left(y\right)=1$ for y≥0 and $\text{Θ}\left(y\right)=0$ for y<0. Eq. 11 follows from Eqs. 8 and 9, assuming $n\left(\vec{x},t\right)$ takes the form in Eq. 10 [36].

In epitope-aligned coordinates, $n\left(\vec{s},t\right)$ and $h\left(\vec{s},t\right)$ travel along a constant velocity vector with nontrivial components along multiple directions (Fig. 2C). Each of these components determines the rate at which the associated epitope evolves.

As derived in [36], the parameters v, σ, and $N_{I}$ are not independently specified. Given the dynamics specified by Eqs. 8 and 9, they are determined by ξ, $N_{h}$, D, m, and $R_{0}$. See Methods for the relevant expressions.

## Results

We first describe static properties that the memory and protection distributions must satisfy to be consistent with the two blunting models, regardless of the transmission dynamics that give rise to them. We then derive the consequences for pathogen evolution: to obtain serial strain replacement, the speed of evolution, the strain diversity, and the time to most recent common ancestor are constrained by an upper bound on the reproductive number $R_{0}$ determined by memory creation, cross-protection, and epitope interactions.

### Blunting neighborhoods

In both the every-epitope model and the any-epitope model, blunting implies a geometric condition that no individual’s memory profile contains specific memory to strains that are too close to one another. The precise definition of “too close” differs between the models.

**Figure 3:**
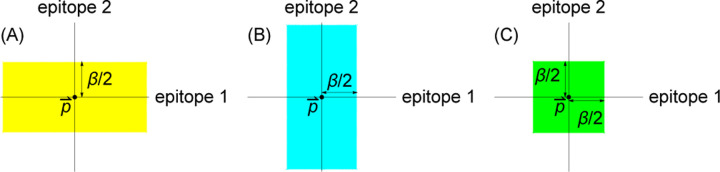
Blunting neighborhoods about reference point $\vec{p}$ for two blunting models, shown in 2*d* antigenic space. (A, B) Blunting neighborhoods in the every-epitope model. (C) Blunting neighborhood in the any-epitope model.

The every-epitope assumption (Eq. 3) implies that for every pair of strains $\vec{s}$, ${\vec{s}}^{\prime }$ in a host’s memory profile 𝒮, the difference $\left|s_{i}-s_{i}^{\prime }\right|$ is at least β in each dimension i=1,…,d. This means that given any reference point $\vec{p}$ in antigenic space, there exists at most one strain $\vec{s}$ in 𝒮 within each of the d stripe-shaped regions defined by the set of points ${\vec{p}}^{\prime }$ such that $\left|p_{i}-p_{i}^{\prime }\right|<\frac{\beta }{2}$ for one value of i between 1 and d inclusive (Fig. 3A,B). Each of these stripes has width β parallel to the *i*th axis and extends infinitely in the remaining $\left(d-1\right)$ axes. We refer to these d regions as the blunting neighborhoods of $\vec{p}$ in the every-epitope model and denote them ${\;}^{\text{E}}{\langle \langle \vec{p}\rangle \rangle }_{\beta }^{i}$.

Eq. 4 implies that in the any-epitope model, for every pair of strains $\vec{s}$, ${\vec{s}}^{\prime }$ in a host’s memory profile 𝒮, the difference $\left|s_{i}-s_{i}^{\prime }\right|$ is at least β in at least one dimension i=1,…,d. This means that given any reference point $\vec{p}$ in antigenic space, there exists at most one strain $\vec{s}$ in 𝒮 within the set of points ${\vec{p}}^{\prime }$ such that $\left|p_{i}-p_{i}^{\prime }\right|<\frac{\beta }{2}$ for all i=1,…,d. Each of these sets of points is a *d*-dimensional cube with side length β centered at $\vec{p}$ (Fig. 3C). We refer to this cube as the blunting neighborhood of $\vec{p}$ in the any-epitope model and denote it ${\;}^{\text{A}}{\langle \langle \vec{p}\rangle \rangle }_{\beta }$.

### Order-dependent protection

**Figure 4:**
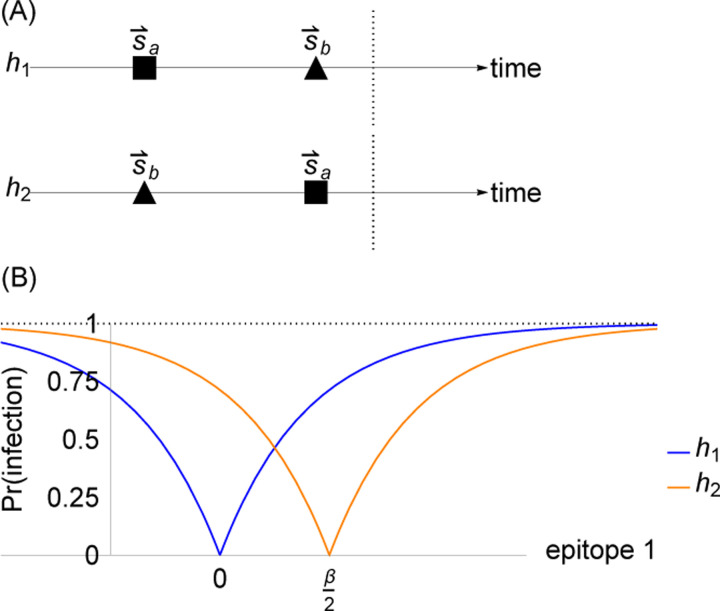
Protection is order-dependent in the blunting model. (A) Infection history of hosts $h_{1}$ and $h_{2}$. (B) Memory profiles and protection distributions of hosts $h_{1}$ and $h_{2}$. Here, $\xi =\frac{\beta }{2}$.

Both proposed blunting models are consistent with a major observation motivating our study of OAS: they imply that individuals’ immunity depends not only on the set of strains to which they have been exposed but also on the order of exposure (Fig. 4) [60, 70].

Consider the simplest case of a pathogen whose evolution takes place in one-dimensional antigenic space. In one dimension, the every-epitope model and the any-epitope model are equivalent. (The generalization to higher dimensions is straightforward for both models.) Two strains are present: one, ${\vec{s}}_{a}$, at the origin and the other, ${\vec{s}}_{b}$, at β/2. Consider two different hosts, $h_{1}$ and $h_{2}$, both of whom have been infected by strains ${\vec{s}}_{a}$ and ${\vec{s}}_{b}$ but in different orders: $h_{1}$ was infected by ${\vec{s}}_{a}$ followed by ${\vec{s}}_{b}$, and $h_{2}$ was infected by ${\vec{s}}_{b}$ followed by ${\vec{s}}_{a}$ (Fig. 4A). Host $h_{1}$ develops specific memory to strain ${\vec{s}}_{a}$ but not to strain ${\vec{s}}_{b}$, whereas host $h_{2}$ develops memory to strain ${\vec{s}}_{b}$ but not ${\vec{s}}_{a}$ (Fig. 4B). Given these memory profiles, the probability that each host is infected by any strain ${\vec{s}}^{\prime }$ is given by

(12) $$\mathrm{Pr}\left(\text{infection by }{\vec{s}}^{\prime }|\text{exposure at }{\vec{s}}^{\prime },h_{1}\right)=\left(1-e^{-\left|s^{\prime }\right|/\xi }\right)$$

(13) $$\mathrm{Pr}\left(\text{infection by }{\vec{s}}^{\prime }|\text{exposure at }{\vec{s}}^{\prime },h_{2}\right)=\left(1-e^{-\left|\frac{\beta }{2}-s^{\prime }\right|/\xi }\right)$$

For example, if host $h_{1}$ is exposed to the strain ${\vec{s}}^{\prime }$ at β, they will be infected with probability $\left(1-e^{-\beta /\xi }\right)$, whereas if host $h_{2}$ is exposed to the same ${\vec{s}}^{\prime }=\beta$, they will be infected with probability $\left(1-e^{-\beta /2\xi }\right)$, a smaller value. Although $h_{1}$ and $h_{2}$ have the same set of exposures in their history, blunting dynamics lead to *h*_1_ having less protection to ${\vec{s}}^{\prime }$ than $h_{2}$ (Fig. 4C). Both blunting models therefore predict order-dependent protection to antigenically diverged strains.

### Immune blind spots

Epidemiological observations indicate that individuals may not develop specific memory against a strain ${\vec{s}}^{\prime }$ even after repeated exposures [60, 71]. We refer to such strains as “immune blind spots” [64]. In both the every-epitope model and the any-epitope model, immune blind spots arise when β is large compared to ξ: the blunting conditions guarantee the existence of points that are too antigenically similar to existing memory to trigger creation of new memory upon exposure but are nonetheless far enough to escape cross-protection from infection.

**Figure 5:**
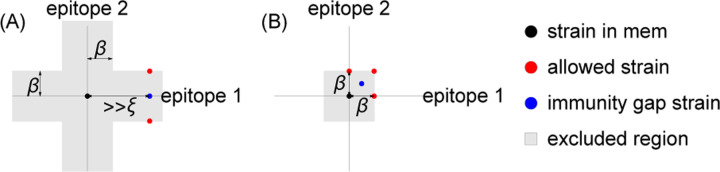
Immune blind spots arise when β is large compared to ξ. Given a memory at the origin, the “allowed strains” are examples of the closest possible strains that could be added to the memory profile. The protection is necessarily low at the immunity blind spot strains regardless of infection history in (A) the every-epitope model and (B) the any-epitope model.

In the every-epitope model, if strain $\vec{s}$ is in a host’s memory profile 𝒮, no strain ${\vec{s}}^{\prime }$ that differs from $\vec{s}$ by a distance less than β in at least one dimension can be in the memory profile. Let ${\vec{s}}^{\prime }$ be a strain with $s_{1}^{\prime }=s_{1}$ and ${\left(\sum_{i=2}^{d}{\left(s_{i}^{\prime }-s_{i}\right)}^{2}\right)}^{1/2}\gg \xi$. The strain ${\vec{s}}^{\prime }$ cannot be added to 𝒮 even if the host is infected by ${\vec{s}}^{\prime }$ at some time in the future. The closest strains to ${\vec{s}}^{\prime }$ that could be added to 𝒮 are the two strains with coordinates $\left(s_{1}\pm \beta ,s_{2}{\;}^{\prime },\ldots ,s_{d}{\;}^{\prime }\right)$. If the blunting distance β is sufficiently large compared to the cross-protection distance ξ, the maximum protection the individual could have at ${\vec{s}}^{\prime }$ is well-approximated by the protection conferred by these two closest strains, even if 𝒮 also contains strains further away. The minimum probability of infection upon exposure to ${\vec{s}}^{\prime }$ is thus approximately ${\left(1-e^{-\beta /\xi }\right)}^{2}$, which approaches 1 for sufficiently large β/ξ. The host thus has an immune blind spot at ${\vec{s}}^{\prime }$ (Fig. 5A).

A similar observation holds for the any-epitope model. An individual with strain $\vec{s}$ in their memory profile 𝒮 cannot have specific memory to any strain ${\vec{s}}^{\prime }$ whose coordinates are all within a distance β of $\vec{s}$. Let ${\vec{s}}^{\prime }$ be the strain with coordinates $s_{i}^{\prime }=s_{i}+\beta /2$ for i=1,…,d. Since $\vec{s}$ is in 𝒮 and ${\vec{s}}^{\prime }$ differs from $\vec{s}$ by less than the blunting distance β in every dimension, ${\vec{s}}^{\prime }$ cannot be in 𝒮. The closest strains to ${\vec{s}}^{\prime }$ that could be in 𝒮 are strains where the *i*th coordinate is either equal to $s_{i}$ or equal to $s_{i}+\beta$ for each i=1,…,d. As in the every-epitope case, for sufficiently large β/ξ, the maximum protection the individual could have at ${\vec{s}}^{\prime }$ is well-approximated by the protection conferred by these closest strains if all these strains were in 𝒮. Including $\vec{s}$, there are 2^*d*^ such strains, each with distance from ${\vec{s}}^{\prime }$ equal to $d^{1/2}\beta /2$. The minimum probability that an individual with $\vec{s}$ in their memory profile is infected by ${\vec{s}}^{\prime }$ upon exposure therefore equals ${\left(1-e^{-d^{1/2}\beta /2\xi }\right)}^{2^{d}}$. This is close to 1 for sufficiently large β≫ξ, meaning that the host has an immune blind spot at ${\vec{s}}^{\prime }$ (Fig. 5B).

### Population-scale memory constraint

Both blunting models imply maximum total numbers of specific memories the population maintains in certain regions of antigenic space. This follows from the existence of blunting neighborhoods. Each host has at most one memory within the blunting neighborhood(s) ${\;}^{\text{E},\text{A}}{\langle \langle \vec{p}\rangle \rangle }_{\beta }^{\left(i\right)}$ of any reference point $\vec{p}$ in antigenic space. (The i index applies to the every-epitope model only.) It follows by taking the sum of memories at each point over all hosts that in any collection of $N_{h}$ non-naive individuals with collective memory distribution $h\left(\vec{s},t\right)$, the total number of memories in each blunting neighborhood of $\vec{p}$ cannot exceed $N_{h}$. In symbols, for both blunting models, we have

(14) $$\int_{{\vec{s}}^{\prime }\in^{\text{E},\text{A}}{\langle \langle \vec{p}\rangle \rangle }_{\beta }^{\left(i\right)}}d{\vec{s}}^{\prime }h\left({\vec{s}}^{\prime },t\right)\leq N_{h}.$$

The every-epitope model and the any-epitope model differ in the regions over which the integral is taken, with the every-epitope model integrating over d infinitely extended stripes to $\vec{p}$, and the any-epitope model integrating over a single *d*-cube to $\vec{p}$ (Fig. 3).

We refer to Eq. 14 as the OAS constraint. It holds in any model of OAS with blunting.

### Limits on successive strain replacement dynamics

We find that the conditions for serial strain replacement are harder to obtain in models that assume blunting (OAS). Only pathogens with a sufficiently low log $R_{0}$ compared to the ratio of the cross-reactivity distance to the blunting distance can exhibit successive strain replacement dynamics in the large-*m* limit.^1^ This bound implies a maximum speed of antigenic evolution that depends on the blunting model.

In the dynamics described by [36], the population builds up a large number of memories in a small interval close to the wavefront. This localized buildup of memory is necessary for maintaining the fitness gradient required to force the infection distribution to move at a constant speed while maintaining its shape. We have shown, however, that the OAS constraint prevents arbitrarily localized buildup of memory. Since the shape of blunting neighborhoods depends on the blunting model, the amount of memory close to the wavefront is also sensitive to specific assumptions about blunting.

**Figure 6:**
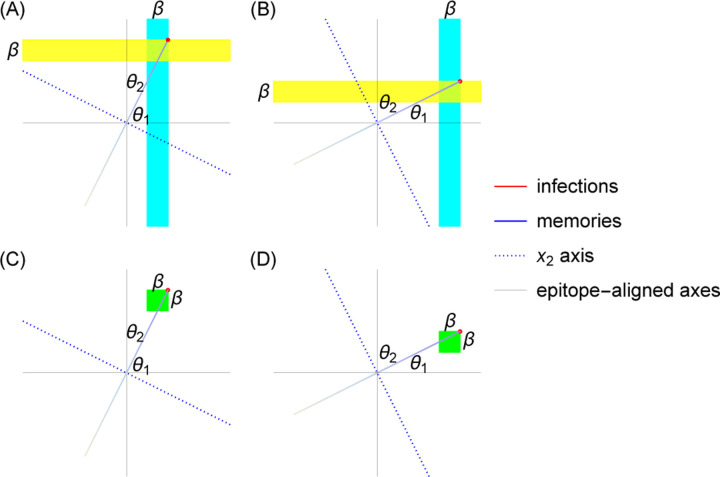
The slowest (fastest) epitope determines the integration bounds in the every- (any-) epitope model. (A, B) The every-epitope model. (A) The blue stripe is the blunting neighborhood that maximizes the number of memories being integrated over. The interval of $x_{1}$ that lies in the blue region has length $\beta /\cos\theta_{1}$. (B) The yellow stripe is the blunting neighborhood that maximizes the number of memories being integrated over. The interval of $x_{1}$ that lies in the yellow region has length $\beta /\cos\theta_{2}$. (C, D) The any-epitope model. The green regions are the blunting neighborhoods that maximize the number of memories being integrated over. The length of the $x_{1}$ interval that lies in the green region equals $\beta /\cos\theta_{2}$ in (C), and $\beta /\cos\theta_{1}$ in (D).

The relevant geometries are shown in Fig. 6. In the successive strain replacement regime, the integral of the memory distribution, $h\left(\vec{s},t\right)$, over a blunting region is always maximized at the wavefront (Methods). The OAS constraint becomes a bound on the value of the integral of memory in the $x_{1}$ direction over an interval whose length ℓ depends on the geometry of the blunting region and the direction of motion of the wave. For the every-epitope model, the length ℓ is determined by the slowest evolving epitope (Fig. 6A,B), whereas for the any-epitope model it is determined by the fastest evolving epitope (Fig. 6C,D).

Let $\theta^{*}$ be the angle between $\vec{v}$ and the axis of the slowest evolving epitope in the every-epitope model and the axis of the fastest evolving epitope in the any-epitope model. The length of the interval over which memory is integrated is $\ell =\frac{\beta }{\cos\theta^{*}}$.

We compute the integral of memory over a blunting region for either blunting model in one step. For any length ℓ, we have

(15) $$\int_{x_{1}^{\prime }=vt-𝓁}^{vt}dx_{1}^{\prime }\left(\frac{mN_{h}}{v\tau }\right)e^{-\frac{\left(vt-x_{1}^{\prime }\right)}{v\tau }}=mN_{h}\left(1-e^{-\ell /v\tau }\right).$$

The OAS constraint requires that this integral equals at most $N_{h}$. This implies $\frac{\beta }{v\tau \cos\theta^{*}}\leq -\log\left(1-\frac{1}{m}\right)$.

As derived in [36], in the traveling wave model with dynamics described by Eqs. 8 and 9, $v\tau =\frac{\xi }{R_{0}^{1/m}-1}$. Algebraic manipulation reveals that the OAS constraint requires that

(16) $$\log R_{0}\leq \frac{\xi \cos\theta^{*}}{\beta }$$

in the large-*m* limit (detailed derivation in Methods). Since $\cos\theta_{i}$ measures how quickly the *i*th epitope evolves compared to the other $\left(d-1\right)$ epitopes, this equation says that successive strain replacement is possible only if $R_{0}$ is not too large compared to the inverse of the blunting distance β in units of the cross-protection distance ξ, modulated by the projection onto the most important epitope for the blunting model of interest.

**Figure 7:**
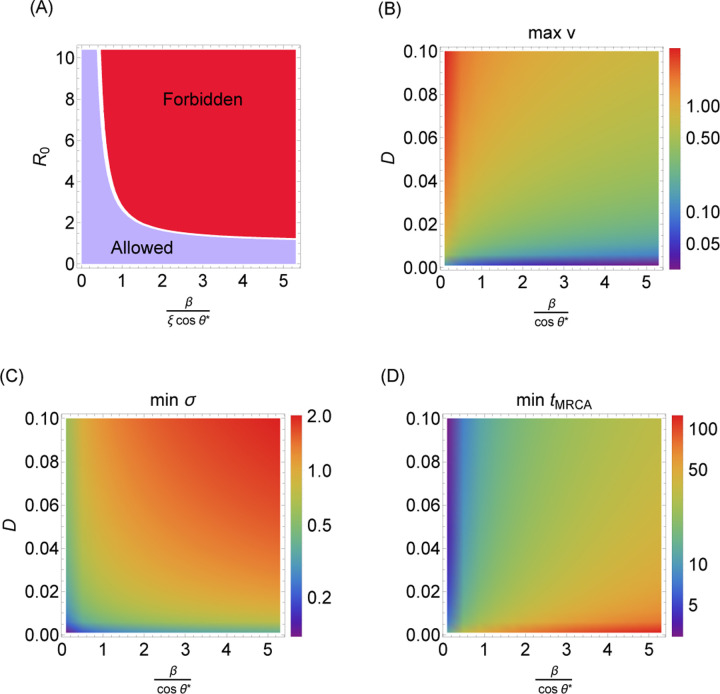
Transmission and dynamical limits implied by the OAS constraint. (A) Strain replacement imposes a maximum value on $R_{0}$. (B) Maximum speed of evolution v (antigenic distance per time measured in infection intervals). (C) Minimum standing diversity σ (antigenic distance). (D) Minimum time to most recent common ancestor, *t*_MRCA_ (measured in infection intervals). D is the diffusion constant. For plotting purposes $N_{h}$ was taken to equal 10^6^ and ξ was taken to equal 1 (B-D).

In the traveling wave model, the cross-protection distance, ξ; the population size, $N_{h}$; the diffusion constant, D; and $R_{0}$ determine derived quantities governing pathogen evolution, including the speed v at which the pathogen evolves, the standard deviation σ of infections in the direction of evolution, and the time to most recent common ancestor, *t*_MRCA_, of cocirculating strains [36] (Methods). The inequality in Eq. 16 implies that in the context of successive strain replacement, blunting leads to ($N_{h}$- and D-dependent) maximum v, minimum σ, and minimum *t*_MRCA_ (Fig. 7).

## Discussion

Many factors have been hypothesized to lead to serial strain replacement, but theoretical tests of these hypotheses have tended to assume that individuals uniformly develop protective strain-specific immunity from infection. We’ve shown that OAS imposes a limitation on the density of population memory in antigenic space, a picture we expect to hold broadly across models of strain evolution. This bound on the number of memories limits the successive strain replacement regime to low-transmissibility pathogens, with “more OAS” corresponding to a tighter bound. Our results imply that observed patterns of serial replacement in nature may be less stable than thought.

This work represents a qualitatively new approach compared to past studies of successive strain replacement. Previous work on waves in antigenic space [36] found that traveling waves solve the model for any set of parameters. Other investigations into the existence of traveling waves in pathogen evolution models [24,72–74] focused on phenomena such as disease treatment [73,74] without including OAS. These models found traveling wave solutions for all $R_{0}>1$ with a *minimum* speed of evolution, as opposed to our result, which implies that OAS imposes a *maximum* speed of evolution. The previous studies that implemented OAS assumed successive strain replacement and examined its impact in age-structured models [64, 66]. Our results are complementary, in that we identify where successive strain replacement is likely to arise in the first place.

By preventing arbitrary buildup of population memory in localized regions of antigenic space, OAS effectively limits the steepness of the fitness landscape that drives evolution to escape immunity, and thus restricts the space of possible infection dynamics. Traveling waves are one example of a dynamical pattern that is subject to this restriction, as they require a dense buildup of memory near the wavefront. The necessary density depends on the characteristic cross-protection distance ξ and the reproduction number $R_{0}$, with smaller ξ and larger $R_{0}$ resulting in more memory near the wavefront. Since the blunting distance β controls the number of memories that are “captured” in the blunting neighborhood at the wavefront, traveling wave dynamics are only consistent for small enough β in relation to $\xi^{-1}$ and $R_{0}$.

The implementation of the OAS bound in terms of blunting neighborhoods also demonstrates an intimate connection between OAS and the rules of memory creation in the presence of multiple evolving epitopes. The geometry of blunting neighborhoods runs parallel to the epitope-aligned axes. By contrast, as long as more than one epitope is evolving, the traveling wave does not travel parallel to any axis. Consequently, the number of memories captured in the blunting neighborhood near the wavefront depends on the speed at which different epitopes evolve. Because the shape of blunting neighborhoods depends on the interactions among epitopes, the slowest evolving epitope determines the upper bound on $R_{0}$ for the every-epitope model, and the fastest evolving epitope determines the bound for the any-epitope model.

The importance of $R_{0}$ and epitopes’ rates of evolution suggests that effective public health measures, including non-pharmaceutical interventions and vaccines, might make pathogens more likely to exhibit successive strain replacement. An immunogenic, well-matched vaccine can deliver a high enough or “long enough” dose of antigen to overcome immune memory, forcing responses to new sites and the creation of strain-specific memory (e.g., [48, 53, 68]). In contrast, at small doses of antigen, immune memory might effectively clear antigen unless every epitope is so antigenically diverged that new strain-specific memory can form. This suggests that population immunity dominated by immunogenic vaccines is likely better approximated by the any-epitope model. Since $\cos\theta^{*}$ is always greater in the any-epitope model than in the every-epitope model, the OAS bound on $R_{0}$ is weaker. In other words, we expect pathogens will be more likely to exhibit successive strain replacement when vaccines can induce broader memory than infections.

One limitation of this work is that the population-level OAS constraint is imposed, rather than allowed to emerge from the model dynamics. Although we expect successive strain replacement models to generically induce memory buildup near the wavefront, we would need to incorporate additional mechanisms into the model for memory dynamics to recapitulate OAS endogenously. We still expect that regimes with a moving wavefront exist, but their functional form may be more complex or even time-dependent. Since all solutions to such a model would satisfy the OAS constraint by design, restrictions on the parameter regimes where successive strain replacement occurs would derive from existence conditions on wavelike solutions to the differential equation rather than consistency conditions on memory at the wavefront. Since in such a model it would be possible for memory to saturate, we hypothesize that a cross-protective, short-term immunity might be required to avoid repeated infections from accumulating, consistent with the conditions in [12, 27]. With dynamics that enforce OAS, it would be possible to characterize evolutionary dynamics in more regimes, e.g., to identify the conditions under which branching occurs. It would be interesting to compare such a study to [6, 34], which construct similar phase diagrams in models without OAS.

There is substantial heterogeneity in antibody-mediated protection against influenza and SARS-CoV-2 in the human population, with individuals showing diversity in their antibody landscapes [39, 65, 75, 76] and the particular epitopes they target, including their sensitivity to different escape mutations [71,77–80]. Some of this heterogeneity arises from differences in initial exposures [71], but there is striking diversity within birth cohorts [65, 76]. Future versions of this model could potentially include different subpopulations, distinguished by their memory repertoires, that are tracked over time and parameterized empirically.

Several assumptions should be reexamined in applying this theory to data, namely, the single blunting cross-protection distance ξ across epitopes, the simple memory creation and cross-protection functions, the single dimension of antigenic space for each epitope, and the artificial dichotomy of the any-epitope and every-epitope models. Quantitative longitudinal data on the specificity and function of adaptive immune repertoire after multiple exposures to diverged antigens can help refine these assumptions.

## Methods

### Details of transmission bound calculation

The general strategy for determining the transmission bound for either blunting model is to impose the OAS constraint over blunting neighborhoods of the appropriate geometry, find the neighborhood over which the integral of the memory distribution is maximized, carry out the integral, and determine the consistency conditions for the inequality to be satisfied.

In the every-epitope model, the ith blunting neighborhood about a point $\vec{p}$ takes the form ${\;}^{E}{\langle \langle \vec{p}\rangle \rangle }_{\beta }^{i}=\left\{\vec{s}:p_{i}\beta /2<s_{i}<p_{i}+\beta /2;-\infty <s_{k}<\infty \text{ for }k\neq i\right\}$. The memory distribution in the successive strain regime falls off exponentially into the past from the wavefront $x_{1}=vt$. While blunting neighborhoods are infinitely extended rectangles parallel to the axes, the direction in which the wave travels is not generally parallel to the axes, so the amount of memory captured in the blunting neighborhoods depends on the direction the wave is traveling. The blunting neighborhood over which the integral of memory is maximized is parallel to the axis of the slowest-evolving epitope, with the wavefront at one of its corners (Fig. 6A,B). The memory captured therefore corresponds to the interval $vt-\frac{\beta }{\cos\left(\theta^{*}\right)}<x_{1}<vt$ in wave-adapted coordinates. The every-epitope OAS constraint simplifies to

(17) $$\int_{x_{1}^{\prime }=vt-\frac{\beta }{\cos\left(\theta^{*}\right)}}^{vt}dx_{1}^{\prime }\left(\frac{mN_{h}}{v\tau }e^{-\left(vt-x_{1}^{\prime }\right)/v\tau }\right)\leq N_{h}$$

In the any-epitope model, the blunting neighborhood about a point $\vec{p}$ takes the form ${\;}^{A}{\langle \langle \vec{p}\rangle \rangle }_{\beta }=\left\{\vec{s}:p_{i}-\beta /2<s_{i}<p_{i}+\beta /2\text{ for }i=1,\ldots ,d\right\}$. These blunting neighborhoods are *d*-dimensional cubes with side length β and sides parallel to the axes. As in the every-epitope model, the amount of memory captured in the blunting neighborhoods depends on the direction the wave is traveling. For the any-epitope geometry, the blunting neighborhood over which the integral of memory is maximized again has the wavefront at one of its corners. This time, however, the length of the interval captured in the neighborhood depends on the direction of the fastest-evolving epitope. The memory captured still corresponds to the interval $vt-\frac{\beta }{\cos\left(\theta^{*}\right)}<x_{1}<vt$ in wave-adapted coordinates (Fig. 6C,D), but now $\theta^{*}$ indicates the fastest-evolving rather than the slowest-evolving direction. The any-epitope OAS constraint simplifies to

(18) $$\int_{x_{1}^{\prime }=vt-\frac{\beta }{\cos\left(\theta^{*}\right)}}^{vt}dx_{1}^{\prime }\left(\frac{mN_{h}}{v\tau }e^{-\left(vt-x_{1}^{\prime }\right)/vt}\right)\leq N_{h}$$

Both of these integrals are of the form $\int_{x_{1}^{\prime }=vt-\ell }^{vt}dx_{1}^{\prime }\left(\frac{mN_{h}}{v\tau }e^{-\left(vt-x_{1}^{\prime }\right)/v\tau }\right)$ with different values of ℓ, so we evaluate this integral and then substitute for ℓ to obtain the relevant inequalities for each blunting model. The inequality simplifies to the condition

(19) $$m\left(1-e^{-\ell /v\tau }\right)\leq 1.$$

This is equivalent to the condition that

(20) $$\frac{\ell }{v\tau }\leq -\log\left(1-\frac{1}{m}\right)$$

The derived quantity vτ can be expressed in terms of the fundamental parameters that characterize the system by manipulating several expressions derived in [36]. The timescale τ equals $mN_{h}/N_{I}$. The incremental fitness gradient along the direction of motion of the wave is analogous to the fitness effect of mutations, s, and equals $\frac{m}{\xi }\left(R_{0}^{1/m}-1\right)$ in the traveling wave system. Marchi et al. argue that s is related to v via the relation $s=\frac{N_{I}}{N_{h}v}$. It follows that

(21) $$\frac{1}{v\tau }=\frac{1}{v}\frac{N_{t}}{mN_{h}}=\frac{s}{m}=\frac{1}{\xi }\left(R_{0}^{1/m}-1\right)$$

so that the inequality in Eq. 20 becomes

(22) $$\frac{\ell }{\xi }\left(R_{0}^{1/m}-1\right)\leq -\log\left(1-\frac{1}{m}\right).$$

The limit as m becomes large of $m\left(R_{0}^{1/m}-1\right)$ is equal to log $R_{0}$, and more generally the following inequality holds for any value of m greater than 1,

(23) $$\log R_{0}<m\left(R_{0}^{1/m}-1\right).$$

Together, Eq. 22 and Eq. 23 imply that the following inequality among the fundamental parameters that characterize transmission and the immune response must hold,

(24) $$\log R_{0}<\frac{\xi }{\ell }\cdot m\log\left(\frac{m}{m-1}\right).$$

In the large-*m* limit, Eq. 24 simplifies to

(25) $$\log R_{0}<\frac{\xi }{\ell }$$

This is the same as the expressions given in the Results, where ℓ equals $\frac{\beta }{\cos\theta^{*}}$.

### Expressions for dynamical quantities in traveling wave model

In this section, we give the expressions for dynamical quantities in the antigenic space model, originally derived in [36]. These correspond to the bounds characterized in Fig. 7, expressed in terms of the fitness increment $s=\frac{m}{\xi }\left(R_{0}^{1/m}-1\right)$:

(26) $$\text{Wave velocity}:\;v=\frac{N_{I}}{N_{h}s}$$

(27) $$=D^{2/3}s^{1/3}{\left(24\;\log\left(N_{I}{\left(Ds^{2}\right)}^{1/3}\right)\right)}^{1/3}$$

(28) $$\text{Wave width}:\;\sigma =\sqrt{\frac{v}{s}}$$

(29) $$\begin{array}{l} \text{Average time to most recent common ancestor}:\\ \;t_{MRCA}\approx \left(1.66\right)\cdot \frac{v}{2Ds} \end{array}$$

Note that the wave velocity is defined implicitly through two equations that determine both v and the number of infected hosts $N_{I}$ in terms of the independent parameters of the system $\left(m,R_{0},\xi ,D,N_{h}\right)$.

## Footnotes

1 We work in the large-*m* limit for simplicity, assuming individuals have many (memory-creating) exposures on average. See Methods, Eq. 24, for the finite-*m* correction, which quickly approaches the large-*m* behavior.
